# Recurrent brief positional vertigo as the initial presentation of medial branch of the posterior inferior cerebellar artery territory cerebellar infarction: a case report

**DOI:** 10.1186/s12883-026-05299-1

**Published:** 2026-08-19

**Authors:** Shaoqing Zhang, Yujing Xie, Zhehao Hu, Yuanyuan Chen, Yongxing Su, Zhengfei Ma

**Affiliations:** 1 Anhui Medical University, Hefei, 230000 Anhui China; 2 Bengbu Medical University, Bengbu, 233000 Anhui China; 3 Suzhou Hospital Affiliated to Anhui Medical University, Suzhou, 234000 Anhui China

**Keywords:** Cerebellar infarction, Episodic vestibular syndrome, Isolated vertigo, Posterior circulation ischemia, Diffusion-weighted magnetic resonance imaging, Case report

## Abstract

**Background:**

Isolated episodic vertigo is usually attributed to peripheral vestibular disorders, but may uncommonly represent posterior circulation ischemia. Brief recurrent positional vertigo caused by medial branch of the posterior inferior cerebellar artery (mPICA) territory infarction is uncommon.

**Case presentation:**

A 63-year-old man with poorly controlled hypertension presented with recurrent brief vertigo triggered mainly by lying down, getting up, or turning over in bed. Initial neurological examination, positional tests, and cranial CT were unremarkable, and BPPV was considered but not confirmed. Persistent attacks followed by spontaneous horizontal nystagmus and mild left-sided dysmetria prompted MRI-DWI, which revealed acute infarction in the medial left cerebellar hemisphere and vermis. After antiplatelet and statin therapy, he remained symptom-free at 1-, 3-, and 6-month follow-up, with an mRS score of 0.

**Conclusions:**

mPICA territory infarction may mimic BPPV as brief recurrent positional vertigo. In older patients with vascular risk factors, persistent or atypical positional vertigo warrants repeated neurological examination and early MRI-DWI.

## Background

Vertigo is a common presenting complaint in both emergency and outpatient settings and remains a frequent diagnostic challenge. Among central vascular events, cerebellar infarction is often overlooked because it can present solely with vertigo or vestibular symptoms in the absence of other focal neurological deficits [1–3]. Cerebellar infarction accounts for only about 2%−3% of all ischemic strokes [3]. Among patients with isolated cerebellar infarction, approximately 10.4% may present with isolated vertigo and imbalance, most commonly in the territory of the medial branch of the posterior inferior cerebellar artery (mPICA) [4]. The mPICA primarily supplies cerebellar structures involved in vestibular control, including the inferior vermis, nodulus/uvula region, flocculus, and medial cerebellar hemisphere [4, 5]. The vestibular nuclei are located in the brainstem and are not part of the cerebellar territory supplied by the mPICA. However, these vestibulocerebellar structures are functionally connected with the brainstem vestibular nuclei and contribute to central vestibular processing. Infarction in the mPICA territory typically presents with persistent vertigo, truncal ataxia, and gait instability [4, 6–8]. Episodic vestibular syndrome (EVS) refers to recurrent, discrete attacks of vertigo or dizziness separated by symptom-free or mildly symptomatic intervals [1]. In this report, “isolated EVS” refers to episodic vertigo without obvious focal neurological deficits at initial presentation. Cases of mPICA territory infarction initially manifesting as isolated EVS are rarely reported, and their diagnostic pitfalls and imaging features remain poorly characterized in the literature [4]. Herein, we report a rare case of mPICA territory cerebellar infarction presenting as isolated EVS in a 63-year-old man with poorly controlled hypertension. This case is noteworthy because the early symptom pattern closely mimicked BPPV, whereas central neurological signs became evident only later in the clinical course.

## Case presentation

A 63-year-old man was admitted on 27 February 2025 with a 12-h history of recurrent episodic vertigo. He developed recurrent vertigo attacks 12 h before admission, mostly triggered by lying down, getting up, or turning over in bed, and occasionally occurring without a clear positional trigger. Each attack lasted approximately 2 min, accompanied by nausea, vomiting, and diaphoresis. No other neurological deficits were present, including limb weakness, numbness, diplopia, dysarthria, or gait instability. During the 12 h before admission, he experienced 3–5 attacks and reported persistent non-spinning dizziness during the interictal period.

The patient had a 10-year history of hypertension, with a peak blood pressure of 185/110 mmHg and poor adherence to antihypertensive treatment.

On admission, his blood pressure was 160/70 mmHg. He was alert and fully oriented, with normal speech. Neurological examination showed no spontaneous or gaze-evoked nystagmus. Bilateral pupils were equal, round, and reactive to light. No ophthalmoplegia, facial weakness, or tongue deviation was detected. Muscle strength was graded 5/5 in all four extremities, with normal muscle tone and intact superficial and deep sensation. The finger-to-nose test, heel-to-shin test, and Romberg test with both eyes open and closed were normal. The Dix–Hallpike test and roll test did not induce vertigo or nystagmus.

Emergency cranial computed tomography (CT) showed no obvious abnormalities. Routine hematologic and biochemical test results were unremarkable.

On admission, a peripheral vestibular disorder, particularly BPPV, was initially considered but not confirmed, although posterior circulation ischemia could not be excluded in view of the patient's vascular risk factor profile.

He initially received symptomatic treatment with betahistine injection. Because a vascular cause could not be excluded, clopidogrel 75 mg once daily and rosuvastatin 10 mg once daily were also started. Despite the initial treatment, recurrent vertigo attacks persisted at a frequency of 7–8 times daily with identical clinical features.

On the second day of admission, close observation during vertigo episodes revealed spontaneous horizontal nystagmus (fast phase to the left), aggravated by left gaze. Due to unavailability of Frenzel glasses or video-oculography, visual fixation suppression could not be fully assessed; however, the nystagmus was observable with eyes open and forward gaze. This finding, combined with later mild dysmetria, supported a central process. Mild dysmetria was noted on the left finger-to-nose test, while the right side was intact.

**Fig. 1:**
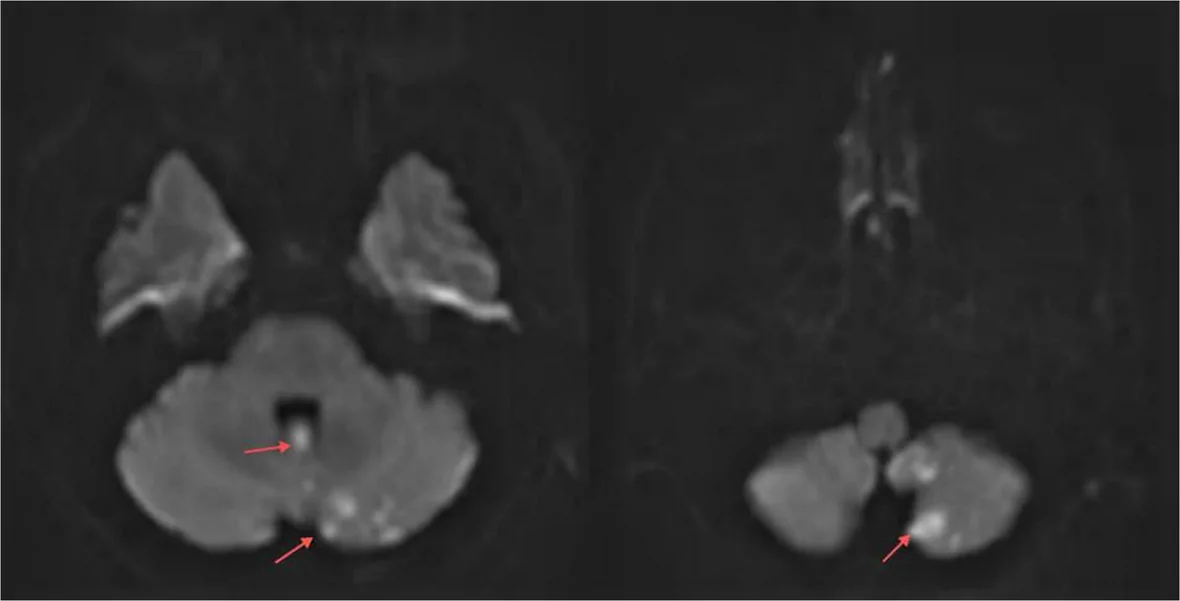
Diffusion-weighted imaging showing hyperintense infarct lesions in the medial left cerebellar hemisphere and cerebellar vermis

**Fig. 2:**
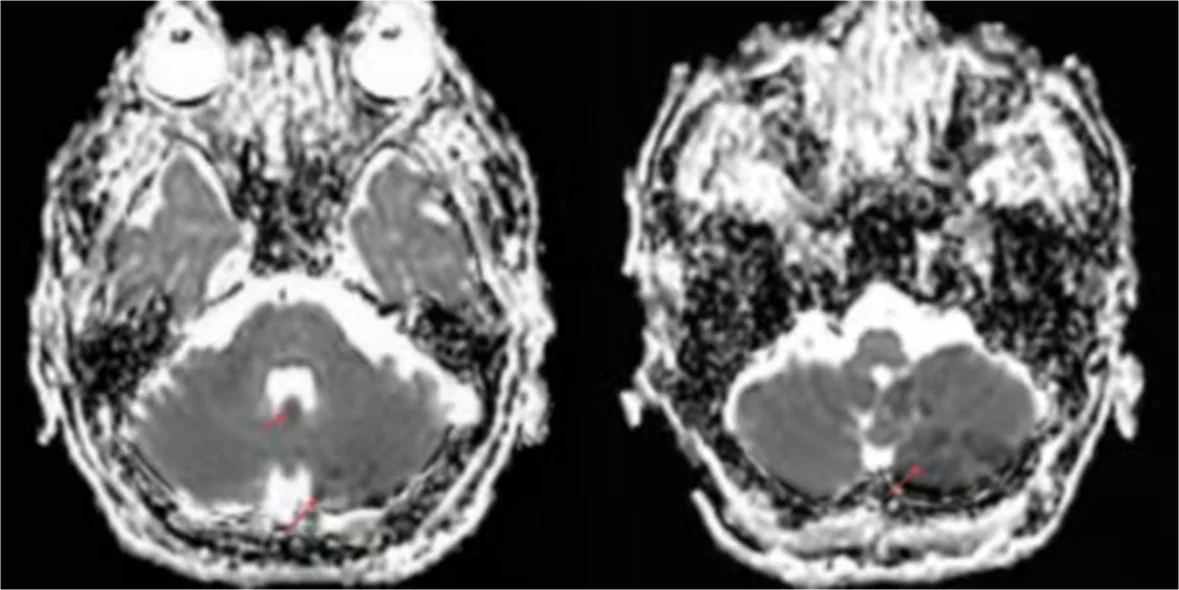
Apparent diffusion coefficient map showing corresponding hypointense lesions in the medial left cerebellar hemisphere and cerebellar vermis

On the third day, brain MRI revealed patchy hyperintensities in the medial left cerebellar hemisphere and cerebellar vermis. The lesions showed markedly high signal on DWI (Fig. 1) and low signal on apparent diffusion coefficient (ADC) maps (Fig. 2) consistent with acute cerebral infarction.

The antiplatelet regimen was adjusted to dual antiplatelet therapy with aspirin 100 mg once daily plus clopidogrel 75 mg once daily. Rosuvastatin was continued for lipid-lowering and plaque stabilization, and edaravone was added. The frequency of vertigo attacks gradually decreased. The patient's condition remained stable.

The final diagnoses were acute cerebellar infarction in the territory of the mPICA and hypertension. The patient was discharged 10 days after admission without residual vertigo, gait instability, or limb ataxia. His modified Rankin Scale score was 0 at discharge.At the 1-, 3-, and 6-month follow-ups, the patient remained free of recurrent vertigo or new neurological symptoms, with a modified Rankin Scale (mRS) score of 0. (Table 1).

**Table 1:** Timeline of the clinical course

Time point	Clinical course
12 h before admission	Recurrent brief positional vertigo began
Day 1	Initial examination, positional tests, and CT were unremarkable; BPPV was considered but not confirmed
Day 2	Spontaneous horizontal nystagmus and mild left-sided dysmetria appeared
Day 3	MRI-DWI confirmed mPICA territory cerebellar infarction
Day 10	Discharged in stable condition
1-, 3-, and 6-month follow-up	No recurrent vertigo or new neurological symptoms; mRS score 0

## Discussion and conclusions

This patient presented with recurrent episodic vertigo as the initial and predominant manifestation of cerebellar infarction. Each attack lasted approximately 2 min, with only dizziness reported during the interictal period. Attacks could be triggered by postural changes, closely mimicking BPPV. However, BPPV became less likely because attacks also occurred without obvious positional triggers and positional tests failed to elicit nystagmus. Given the patient's age and hypertension as a vascular risk factor, posterior circulation transient ischemic attack (TIA) was also considered. Nevertheless, cranial MRI ultimately confirmed acute infarction in the territory of the left medial branch of the mPICA.

The relationship between the present case and BPPV should be interpreted cautiously. BPPV was initially considered because the attacks were brief and frequently triggered by changes in head or body position. However, BPPV was not confirmed because neither the Dix-Hallpike test nor the supine roll test provoked typical positional vertigo or canal-specific positional nystagmus. This presentation is therefore better described as pseudo-BPPV-like rather than definite BPPV [8].

The spontaneous horizontal nystagmus observed on hospital day 2 may result from transient dysfunction of vestibulocerebellar structures (nodulus/uvula/flocculus) in the mPICA territory, disrupting their connections to brainstem vestibular nuclei and ocular motor pathways. This finding, together with mild left-sided dysmetria, further supported a central vestibular process, although bedside observation without Frenzel glasses or video-oculography limited precise characterization of visual fixation suppression.

It should also be distinguished from definite central positional vertigo, in which positional maneuvers usually provoke central positional nystagmus (such as persistent downbeat, purely vertical or torsional, direction-changing, or non-fatigable nystagmus). In the present case, positional testing failed to elicit either peripheral or central positional nystagmus, and the central nature of the disorder became evident only later with the appearance of spontaneous horizontal nystagmus [8].

The typical clinical manifestations of cerebellar infarction depend on the affected vessel and infarct extent. PICA territory infarction involving the dorsolateral medulla oblongata presents as Wallenberg's syndrome, characterized by vertigo, nausea and vomiting, nystagmus, ipsilateral cerebellar ataxia, ipsilateral Horner's syndrome, ipsilateral impaired facial pain and temperature sensation, and contralateral impaired limb pain and temperature sensation [3, 4]. When the infarction is confined to the cerebellum in the mPICA territory without medullary involvement, the main manifestations are symptoms and signs of vestibular impairment, namely vertigo, gait instability, truncal ataxia and lateropulsion, with minimal or absent limb ataxia [4, 6, 8]. Anatomically, the mPICA primarily supplies cerebellar structures, including the inferior vermis, nodulus/uvula region, flocculus, and medial cerebellar hemisphere. The vestibular nuclei are located in the brainstem and are not part of the cerebellar territory supplied by the mPICA. However, these vestibulocerebellar structures have close afferent and efferent connections with the brainstem vestibular nuclei and participate in central vestibular processing. Therefore, mPICA territory infarction may produce vertigo by disrupting vestibulocerebellar–brainstem vestibular network connections, even without direct infarction of the vestibular nuclei [4, 5, 8].

### Review of previously reported similar cases

To place this case in context, we performed a focused literature review of previously reported cases and studies of cerebellar or posterior circulation ischemia presenting with isolated episodic vertigo, brief positional vertigo, posture-related symptoms, or peripheral vestibular-like manifestations. PubMed and Google Scholar were searched up to April 2026 using combinations of the terms “cerebellar infarction”, “PICA”, “medial PICA”, “episodic vertigo”, “positional vertigo”, “BPPV”, and “isolated vestibular syndrome”. Only information explicitly reported in the original articles was extracted, and unavailable or unclear items were marked as NA. The relevant studies are summarized in Table 2.

**Table 2:** Relevant reports and studies of cerebellar or posterior circulation ischemia presenting with isolated, episodic, positional, or peripheral vestibular-like symptoms

Study	Design/cases	Main presentation	Initial diagnostic pitfall	Bedside findings	Lesion/territory	Key relevance to present case
Lee et al., 2006[4]	Consecutive acute-stroke registry study; 240 MRI-confirmed isolated cerebellar infarctions; 25 pseudo-vestibular neuritis cases	Isolated prolonged spontaneous vertigo with nystagmus and imbalance	Mimicked vestibular neuritis/pseudo-vestibular neuritis	Normal head thrust in all mPICA cases; gaze-evoked nystagmus and postural instability were common	PICA territory in 24/25 cases, mostly mPICA; nodulus commonly involved	Shows that mPICA/PICA infarction can mimic peripheral vestibular disease, but mainly as prolonged spontaneous vertigo rather than brief recurrent positional vertigo
Casani et al., 2013[7]	Case series with chart review; 11 pseudo-acute peripheral vertigo cases	Sudden rotational vertigo with nausea/vomiting; vertigo lasted > 72 h in 7 cases	Mimicked acute peripheral vertigo/vestibular neuritis	Initial neurological examination and CT were negative; spontaneous nystagmus in all cases; delayed focal signs in 4 cases	PICA territory in 8 cases; anterior inferior cerebellar artery territory in 1 case; cerebellar hemispheric involvement in 2 cases	Supports that cerebellar infarction can initially mimic peripheral vertigo and show delayed neurological signs, but the presentation was mainly prolonged acute vertigo
Fan et al., 2023[9]	Case report; *n* = 1	Recurrent positional diplopia triggered by standing/walking and relieved by lying down; later dizziness, vertigo, nausea/vomiting and gait instability	NA	Bedside positional tests negative; central nystagmus, ocular tilt findings, Horner sign, crossed sensory signs and left ataxia were reported	PICA territory infarction involving the left medulla and cerebellar vermis; severe bilateral vertebral artery stenosis	Shows that posterior circulation/PICA ischemia may present with recurrent posture-related transient symptoms, but the main prodromal symptom was diplopia rather than isolated vertigo
Tong et al., 2020[10]	Retrospective cohort study; 181 ischemic acute vestibular syndrome events; 68 DWI-positive acute infarcts and 113 DWI-negative ischemic events	Acute or episodic vestibular syndrome caused by ischemic events was present in 41/68 DWI-positive cases	NA	Head motion intolerance, gait unsteadiness and nausea/vomiting were common; spontaneous nystagmus in 9/68; focal signs in 22/68	DWI-positive infarcts involved anterior circulation artery in 29/68, posterior circulation artery in 28/68, and both in 11/68; main sites were insular cortex and posterior thalamus	Supports that ischemic stroke can manifest as EVS, but it is not specific to cerebellar or PICA territory infarction
Present case	Case report; *n* = 1	Recurrent brief positional vertigo lasting about 2 min, triggered by lying down, getting up or turning over in bed	BPPV was considered but not confirmed	Initial neurological examination,Dix–Hallpike, roll test and CT were unremarkable; horizontal nystagmus and mild left dysmetria appeared later	Medial left cerebellar hemisphere and vermis; mPICA territory	Adds a rare pseudo-BPPV-like pattern with brief recurrent positional vertigo, initially negative positional tests and delayed central signs

As shown in Table 2, previously reported cerebellar or posterior circulation ischemic events most commonly presented as prolonged spontaneous vertigo mimicking vestibular neuritis or acute peripheral vertigo. Lee et al. [4] and Casani et al. [7] mainly described prolonged acute vertigo with nystagmus and imbalance, often associated with delayed neurological signs. Fan et al. [9] reported posture-related recurrent diplopia caused by PICA territory infarction, supporting that posterior circulation ischemia may present with recurrent posture-dependent symptoms. Tong et al. [10] further showed that ischemic stroke can manifest as EVS, although the lesion distribution was not limited to the cerebellum or PICA territory. Compared with these reports, the present case is distinctive because the attacks were brief, recurrent, and position-triggered, closely resembling BPPV, while initial positional testing was negative and central neurological signs appeared only later.

Several mechanisms may account for the recurrent episodic symptoms in this case. The attacks may represent transient ischemic events in the vertebrobasilar or mPICA territory preceding completed infarction [2, 11], or fluctuating symptoms of an evolving cerebellar infarct. The progression from recurrent brief position-triggered vertigo on admission to spontaneous horizontal nystagmus and mild left-sided dysmetria over the next 48 h suggests an evolving posterior circulation ischemic process rather than a purely benign positional disorder. The position-triggered nature of the attacks may reflect transient dysfunction of vestibulocerebellar structures within the mPICA territory during changes in head or body position [4, 8]. Hemodynamic fluctuation is a plausible explanation, particularly in patients with vascular risk factors, but recurrent microembolism or vasospasm cannot be excluded.

The present case was highly susceptible to misdiagnosis as a peripheral vestibular disorder during the early stage because of the brief episodic attacks, positional triggers, absence of focal neurological and positional tests, and unremarkable emergency CT. Several clues should raise suspicion for a central lesion in similar patients. First, vascular risk factors remain important, particularly in middle-aged or older patients. Second, nystagmus should be reassessed repeatedly, because central nystagmus may become evident only during the clinical course. Third, even subtle cerebellar signs, such as the mild dysmetria observed in this patient, should prompt reconsideration of a central lesion. These clinical clues are particularly important in episodic presentations, in which bedside vestibular tests may have limited diagnostic value [12].

Imaging examination is critical for the diagnosis of cerebellar infarction. Cranial CT has low sensitivity for posterior fossa lesions, with a missed diagnosis rate of up to 22% at initial presentation. Cranial MRI-DWI is the gold standard for the diagnosis of acute cerebellar infarction, but the false-negative rate within 24 h of symptom onset can reach 20% [3, 13]. Pelletier et al. [14] noted that CT, computed tomography angiography (CTA), and CT perfusion have limited sensitivity for posterior circulation stroke, and that even MRI may miss some posterior circulation strokes in the early stage. A review by De Cocker et al. [13] also emphasized the problem of false-negative findings on early MRI in cerebellar infarction. Therefore, for patients with high clinical suspicion of posterior circulation ischemia but negative initial MRI, repeat MRI after 2 to 3 days should be performed if necessary.

Because cerebrovascular imaging was not performed due to the patient’s physical condition and limited cooperation during hospitalization, the precise vascular mechanism underlying the infarction could not be determined, and TOAST classification remained uncertain. Nevertheless, based on the clinical presentation, CT and MRI findings, and the transient, recurrent positional nature of the symptoms, transient ischemic events, hemodynamic disturbance in the PICA territory, or microembolic phenomena may be possible mechanisms. This limitation should be acknowledged, and future similar cases should incorporate vascular imaging when feasible to better define the underlying vascular pathology.

After the diagnosis was established, the patient received antiplatelet and statin therapy, and his symptoms gradually improved. Previous reports suggest that cerebellar infarction in the mPICA territory presenting with isolated vertigo may be associated with a relatively favorable short-term outcome [4]. However, in the present case, follow-up evaluations up to 6 months confirmed sustained symptom resolution with a modified Rankin Scale (mRS) score of 0. Longer-term monitoring (beyond 12 months) is still warranted to assess the risk of recurrence. In addition, although no clinical deterioration occurred during hospitalization, delayed complications of cerebellar infarction, including progressive stroke and brainstem compression caused by cerebellar edema, should be closely monitored.

The added value of this case lies in demonstrating that mPICA territory cerebellar infarction may present with a pseudo-BPPV-like pattern of brief recurrent position-triggered vertigo, initially negative positional testing, and delayed central signs. Brief recurrent positional vertigo does not exclude posterior circulation ischemia in middle-aged or older patients with vascular risk factors. Repeated neurological examination and early MRI-DWI are warranted when symptoms persist or central signs emerge.

## Data Availability

The data relevant to this case report are included within the article. Requests for additional information may be directed to the corresponding author.

## Abbreviations

mPICA: Medial branch of the posterior inferior cerebellar artery
PICA: Posterior inferior cerebellar artery
BPPV: Benign paroxysmal positional vertigo
CT: Computed tomography
MRI: Magnetic resonance imaging
DWI: Diffusion-weighted imaging
ADC: Apparent diffusion coefficient
TIA: Transient ischemic attack
CTA: Computed tomography angiography
mRS: Modified Rankin Scale
EVS: Episodic vestibular syndrome
